# (3a*S*,4*R*,5*R*,6*S*,7a*R*)-4,5-Di­bromo-2-[4-(tri­fluoro­meth­yl)phen­yl]-2,3,3a,4,5,6,7,7a-octa­hydro-3a,6-ep­oxy-1*H*-isoindol-1-one: crystal structure and Hirshfeld surface analysis

**DOI:** 10.1107/S2056989021003200

**Published:** 2021-04-09

**Authors:** Dmitriy F. Mertsalov, Kseniia A. Alekseeva, Magrycheva S. Daria, Maxim E. Cheshigin, Sevim Türktekin Çelikesir, Mehmet Akkurt, Mikhail S. Grigoriev, Sixberth Mlowe

**Affiliations:** a Department of Organic Chemistry, Peoples’ Friendship University of Russia (RUDN University), 6 Miklukho-Maklaya St., 117198, Moscow, Russian Federation; b Department of Physics, Faculty of Sciences, Erciyes University, 38039 Kayseri, Turkey; c Frumkin Institute of Physical Chemistry and Electrochemistry, Russian Academy of Sciences, Leninskiy prospect 31-4, Moscow 119071, Russian Federation; d University of Dar es Salaam, Dar es Salaam University College of Education, Department of Chemistry, PO Box 2329, Dar es Salaam, Tanzania

**Keywords:** crystal structure, ep­oxy­iso­indole group, hydrogen bond, halogen bond, non-covalent inter­actions, Hirshfeld surface analysis

## Abstract

The asymmetric unit of the title compound, C_15_H_12_Br_2_F_3_NO_2_, consists of two crystallographically independent mol­ecules. In both mol­ecules, the pyrrolidine and tetra­hydro­furan rings adopt an envelope conformation. In the crystal, mol­ecule pairs generate centrosymmetric rings with *R* ^2^_2_(8) motifs linked by C—H⋯O hydrogen bonds. These pairs of mol­ecules form a tetra­meric supra­molecular motif, leading to mol­ecular layers parallel to the (100) plane by C—H⋯π and C—Br⋯π inter­actions. Inter­layer van der Waals and inter­halogen inter­actions stabilize mol­ecular packing. The F atoms of the CF_3_ groups of both mol­ecules are disordered over two sets of sites with refined site occupancies of 0.60 (3)/0.40 (3) and 0.640 (15)/0.360 (15). The most important contributions to the surface contacts of both mol­ecules are from H⋯H (23.8 and 22.4%), Br⋯H/H⋯Br (18.3 and 12.3%), O⋯H/H⋯O (14.3 and 9.7%) and F⋯H/H⋯F (10.4 and 19.1%) inter­actions, as concluded from a Hirshfeld surface analysis.

## Abstract 2

In the crystal structure, mol­ecule pairs generate rings with 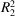
(8) motifs by dimeric C—H⋯O hydrogen bonds. These pairs of mol­ecules form mol­ecular layers parallel to the (100) plane by C—H⋯π and C—Br⋯π inter­actions. Inter­layer van der Waals inter­actions stabilize the mol­ecular packing.

## Chemical context   

Iso­indoles are important structural units in many natural products and are widely used as drugs and as building-blocks for the construction of new N-containing heterocyclic compounds and functional materials (Nadirova *et al.*, 2019 ▸; Zubkov *et al.*, 2011 ▸, 2014 ▸, 2018 ▸). The biological and physical properties of N-heterocycles are dependent on the attached functional groups (Grudova *et al.*, 2020 ▸; Zaytsev *et al.*, 2017 ▸, 2019 ▸, 2020 ▸; Asgarova *et al.*, 2019 ▸; Khalilov *et al.*, 2011 ▸; Yin *et al.*, 2020 ▸). Thus, the functionalization of iso­indole moieties with non-covalent bond donor/acceptor sites can improve their biological and photophysical properties as well as coordination ability (Wicholas *et al.*, 2006 ▸).

On the other hand, non-covalent inter­actions, such as hydrogen, aerogen, halogen, chalcogen, pnictogen, tetrel and icosa­gen bonds, as well as *n*–π*, *π–π* stacking, *π*–cation, *π*–anion and hydro­phobic inter­actions have also attracted much attention recently and have been demonstrated to play a prominent role in synthesis, catalysis, supra­molecular chemistry, mol­ecular recognition, biological systems and functional materials (Asadov *et al.*, 2016 ▸; Gurbanov *et al.*, 2017 ▸, 2018 ▸, 2020 ▸; Karmakar *et al.*, 2017 ▸; Kopylovich *et al.*, 2011 ▸; Ma *et al.*, 2017*a* ▸,*b* ▸; 2020 ▸; Mahmudov *et al.*, 2010 ▸, 2012 ▸, 2013 ▸, 2019 ▸, 2020 ▸; Mizar *et al.*, 2012 ▸; Sutradhar *et al.*, 2015 ▸, 2016 ▸). Halogen bonding is a rather spread phenomenon since halogen atoms or ions can form short non-bonded contacts with electron acceptors, electron donors or be inter­connected due to anisotropic charge distribution in halogen atoms (Afkhami *et al.*, 2017 ▸; Maharramov *et al.*, 2018 ▸, 2019 ▸; Mahmoudi *et al.*, 2017 ▸, 2019 ▸; Shixaliyev *et al.*, 2014 ▸). In fact, attachment of iso­indoles with non-covalent bond donor or acceptor sites can affect their supra­molecular arrangements significantly (Gurbanov *et al.*, 2021 ▸).
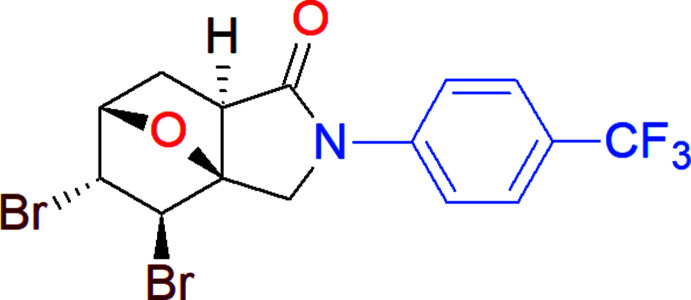


**Figure 1:**
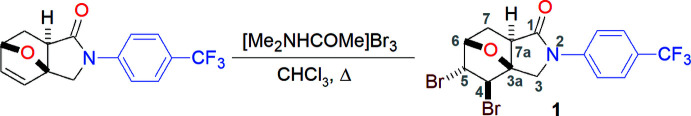
Synthesis of (3a*S*,4*R*,5*R*,6*S*,7a*R*)-4,5-di­bromo-2-[4-(tri­fluoro­meth­yl)phen­yl]-2,3,3a,4,5,6,7,7a-octa­hydro-3a,6-ep­oxy-1*H*-isoindol-1-one (**1**).

In a continuation of our work in this direction, we have functionalized a new iso­indole, (3a*S*,4*R*,5*R*,6*S*,7a*R*)-4,5-di­bromo-2-[4-(tri­fluoro­meth­yl)phen­yl]-2,3,3a,4,5,6,7,7a-octa­hydro-3a,6-ep­oxy-1*H*-isoindol-1-one (**1**; Fig. 1 ▸), which provides C—Br⋯π halogen bonds as well as C—H⋯O and C—H⋯π types of inter­molecular hydrogen bonds.

## Structural commentary   

**Figure 2:**
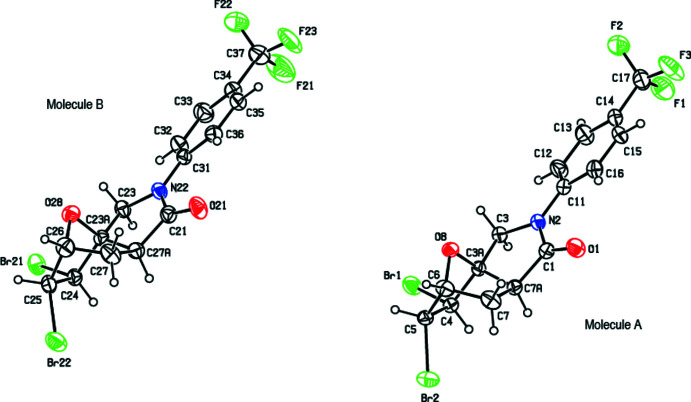
The two mol­ecules (*A* and *B*) in the asymmetric unit of the title compound with displacement ellipsoids for the non-hydrogen atoms drawn at the 30% probability level. Hydrogen atoms are shown as spheres of arbitrary radius. The minor components of the disordered CF_3_ groups were omitted for clarity.

**Figure 3:**
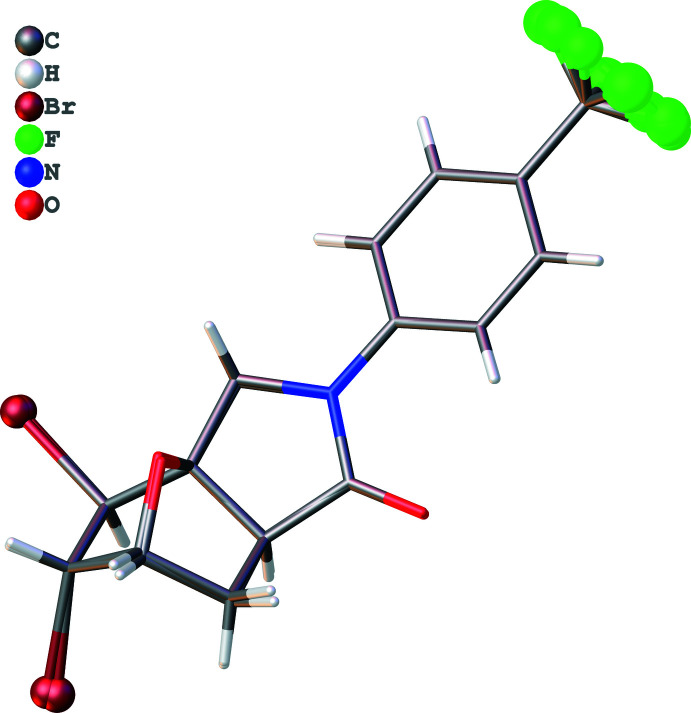
Overlay image (*OLEX2*; Dolomanov *et al.*, 2009 ▸) of the two mol­ecules (*A* and *B*) in the asymmetric unit of the title compound.

The asymmetric unit of the title compound (Fig. 2 ▸) contains two crystallographically mol­ecules of similar shape, hereafter referred to as mol­ecules *A* (including atom C1) and *B* (including atom C21). The conformational differences between mol­ecules *A* and *B* are highlighted in an overlay diagram shown in Fig. 3 ▸. The r.m.s. deviation of the overlay between the mol­ecules *A* and *B* is 0.278 Å.

In both mol­ecules *A* and *B*, the pyrrolidine rings (N2/C1/C3/C3*A*/C7*A* and N22/C21/C23/C23*A*/C27*A*), tetra­hydro­furan rings (O8/C3*A*/C4–C6, O8/C3*A*/C6/C7/C7*A* and O28/C23*A*/C24–C26, O28/C23*A*/C26/C27/C27*A*) and six-membered rings (C3*A*/C4–C7/C7*A* and C23*A*/C24–C27/C27*A*), which generate ep­oxy­iso­indole moieties (O8/ N2/C1/C3/C3*A*/C4–C7/C7*A* and O28/ N22/C21/C23/C23*A*/C24–C27/C27*A*), are puckered. In mol­ecules *A* and *B*, both tetra­hydro­furan rings adopt an envelope conformation with puckering parameters (Cremer & Pople, 1975 ▸) *Q*(2) = 0.580 (3) Å, φ(2) = 176.3 (4)° for *A* (O8/C3*A*/C4–C6), *Q*(2) = 0.547 (3) Å, φ(2) = 357.4 (4)° for *A* (O8/C3*A*/C6/C7/C7*A*), and *Q*(2) = 0.580 (3) Å, φ(2) = 180.3 (4)° for *B* (O28/C23*A*/C24–C26) and *Q*(2) = 0.554 (3) Å, φ(2) = 354.2 (4)° for *B* (O28/C23*A*/C26/C27/C27*A*). The five-membered pyrrolidine rings also exhibit an envelope conformation, with a maximum deviation from the mean plane of 0.165 (3) Å at C3*A* [puckering parameters *Q*(2) = 0.262 (4) Å, φ(2) = 281.8 (8)°] for mol­ecule *A* and 0.156 (3) Å at C23*A* [puckering parameters *Q*(2) = 0.248 (4) Å, φ(2) = 291.3 (8)°] for mol­ecule *B*. In both mol­ecules, the six-membered ring has a boat conformation [*Q*_T_ = 0.925 (4) Å, θ = 92.2 (2)°, φ = 180.5 (2)° for mol­ecule *A*; *Q*_T_ = 0.924 (4) Å, θ = 91.7 (2)°, φ = 177.1 (2)° for mol­ecule *B*].

## Supra­molecular features   

**Figure 4:**
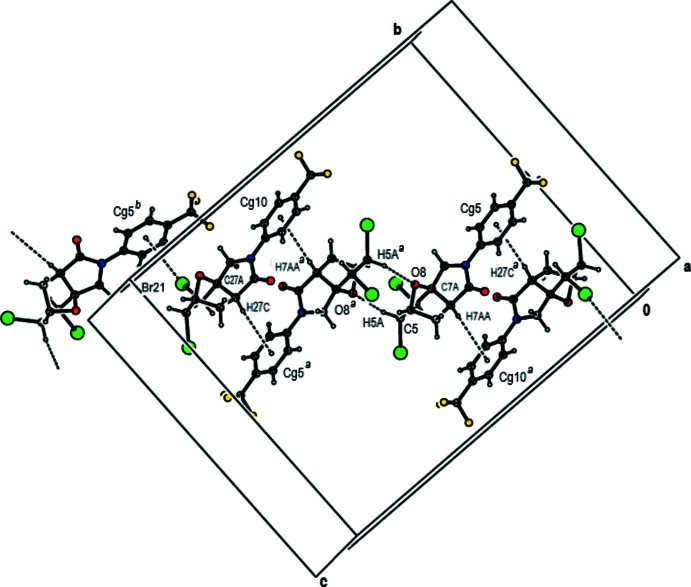
A view of the inter­molecular C—H⋯O hydrogen bonds and C—H⋯π and C—Br⋯π inter­actions in the unit cell of the title compound. Only the major components of the disordered CF_3_ groups are shown. [Symmetry codes: (*a*) 1 − *x*, 1 − *y*, 1 − *z*; (*b*) − 1 + *x*, 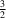 − *y*, 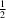 + *z*].

**Figure 5:**
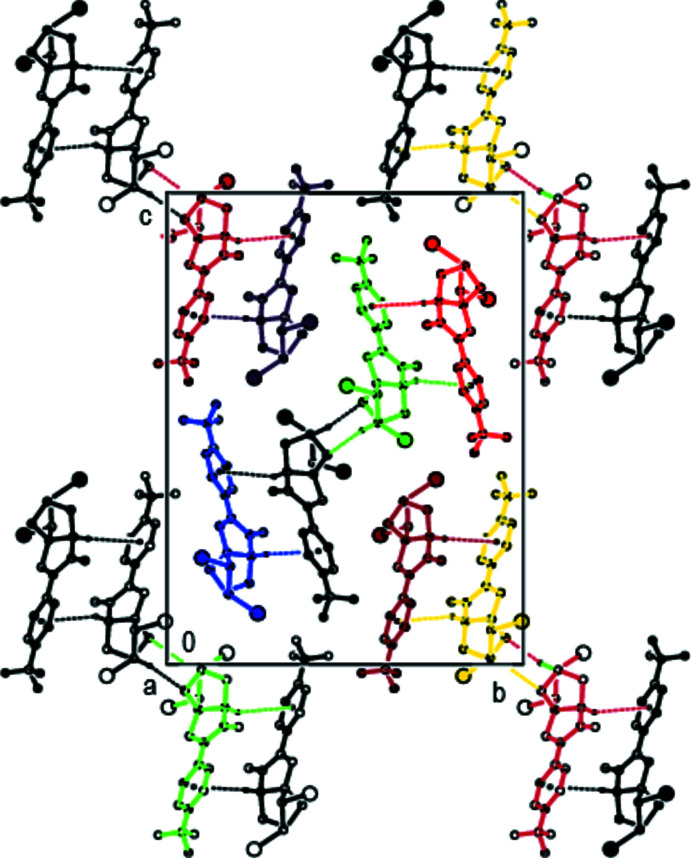
A view of the mol­ecular packing of the title compound along the *a* axis. Only the major components of the disordered CF_3_ groups are shown.

In the crystal, mol­ecules generate centrosymmetric dimers described by 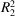
(8) motifs (Bernstein *et al.*, 1995 ▸) by C—H⋯O hydrogen bonds (Table 1 ▸). These pairs of mol­ecules form a tetra­meric supra­molecular motif, by self-complementary C—H⋯π connections (Fig. 4 ▸). Additionally, these building units are self-assembled *via* C—Br⋯π inter­actions, generating a two-dimensional supra­molecular network parallel to the (100) plane (Fig. 5 ▸). Inter­layer van der Waals and inter­halogen inter­actions stabilize mol­ecular packing.

## Hirshfeld surface analysis   

**Figure 6:**
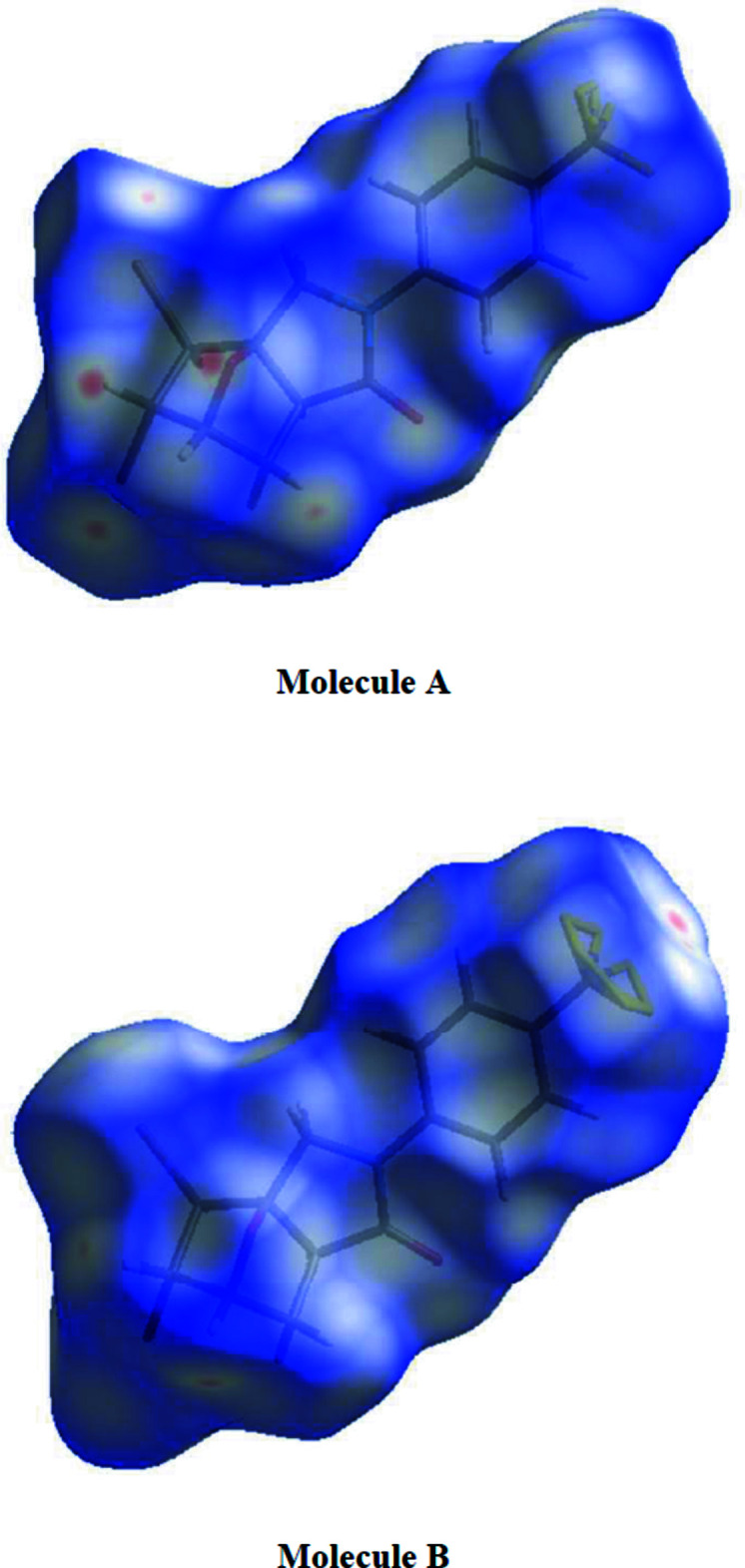
Hirshfeld surfaces of mol­ecules *A* and *B* of the title compound mapped with *d*_norm_.

**Figure 7:**
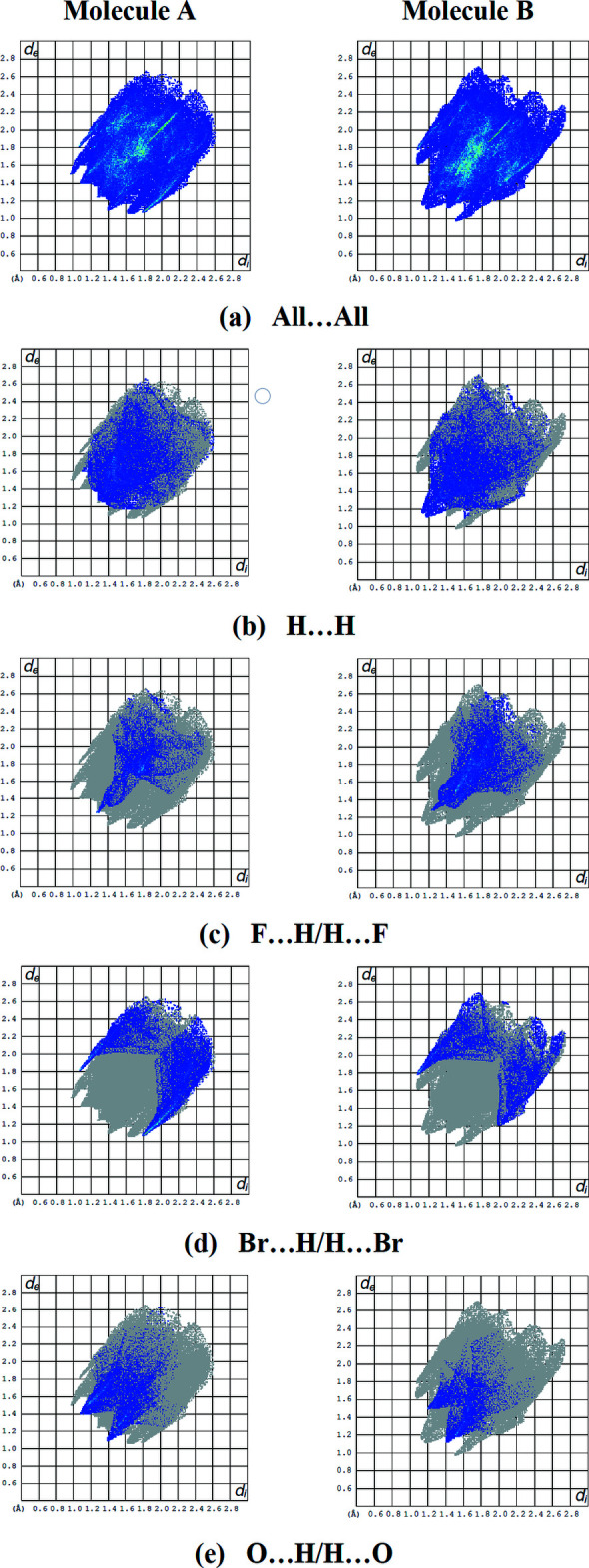
The two-dimensional fingerprint plots for mol­ecules *A* and *B* of the title compound, showing (*a*) all inter­actions, and delineated into (*b*) H⋯H, (*c*) F⋯H/H⋯F, (*d*) Br⋯H/H⋯Br and (*e*) O⋯H/H⋯O inter­actions [*d*_e_ and *d*_i_ represent the distances from a point on the Hirshfeld surface to the nearest atoms outside (external) and inside (inter­nal) the surface, respectively].

For both mol­ecules *A* and *B*, the inter­molecular inter­actions (Table 2 ▸) were qu­anti­fied using Hirshfeld surface analysis (Spackman & Jayatilaka, 2009 ▸) and the associated two-dimensional fingerprint plots (McKinnon *et al.*, 2007 ▸) generated. The calculations and visualization were performed using *CrystalExplorer17* (Turner *et al.*, 2017 ▸). Fig. 6 ▸ shows the Hirshfeld surface of the title compound mapped over *d*_norm_ in a fixed color scale of −0.2089 (red) to +1.1825 (blue) arbitrary units for mol­ecule *A* and −0.2105 (red) to +1.2372 (blue) arbitrary units for mol­ecule *B*, where the red spots indicate the inter­molecular contacts shorter than the van der Waals separations. Fig. 7 ▸ shows the full two-dimensional fingerprint plot (Fig. 7 ▸ *a*) and those delineated into the major contacts: H⋯H (23.8% for mol­ecule *A* and 22.4% for mol­ecule *B*, Fig. 7 ▸ *b*) inter­actions are the major factor in the crystal packing with Br⋯H/H⋯Br (18.3% for mol­ecule *A* and 12.3% for mol­ecule *B*, Fig.7*c*), O⋯H/H⋯O (14.3% for mol­ecule *A* and 9.7% for mol­ecule *B*, Fig. 7 ▸ *d*) and F⋯H/H⋯F (10.4% for mol­ecule *A* and 19.1% for mol­ecule *B*, Fig. 7 ▸ *e*) inter­actions representing the next highest contributions. The percentage contributions of other weak inter­actions are listed in Table 3 ▸. The fact that the same inter­actions make different contributions to the HS mol­ecules *A* and *B* can be attributed to the different mol­ecular environments of the A and B mol­ecules in the crystalline structure.

## Database survey   

A search of the Cambridge Structural Database (CSD version 5.40, update of September 2019; Groom *et al.*, 2016 ▸) for structures having the ep­oxy­iso­indole moiety gave eight hits that closely resemble the title compound, *viz.* 4,5-di­bromo-6-methyl-2-phenyl­hexa­hydro-3a,6-ep­oxy­isoindol-1(4*H*)-one (IMUBIE; Mertsalov *et al.*, 2021*a* ▸), 2-benzyl-4,5-di­bromo­hexa­hydro-3a,6-ep­oxy­isoindol-1(4*H*)-one (OMEMAX; Mertsalov *et al.*, 2021*b* ▸), (3a*R*,6*S*,7a*R*)-7a-chloro-2-[(4-nitrophen­yl)sulfon­yl]-1,2,3,6,7,7a-hexa­hydro-3a,6-ep­oxy­iso­indole (AGONUH; Temel *et al.*, 2013 ▸), (3a*R*,6*S*,7a*R*)-7a-chloro-6-methyl-2-[(4-nitro­phen­yl)sulfon­yl]-1,2,3,6,7,7a-hexa­hydro-3a,6-ep­oxy­iso­indole (TIJMIK; Demircan *et al.*, 2013 ▸), 5-chloro-7-methyl-3-[(4-methyl-phen­yl)sulfon­yl]-10-oxa-3-aza­tri­cyclo­[5.2.1.01,5]dec-8-ene (YAXCIL; Temel *et al.*, 2012 ▸), (3a*R*,6*S*,7a*R*)-7a-bromo-2-[(4-methyl­phen­yl)sulfon­yl]-1,2,3,6,7,7a-hexa­hydro-3a,6-ep­oxy­iso-indole (UPAQEI; Koşar *et al.*, 2011 ▸), (3a*R*,6*S*,7a*R*)-7a-bromo-2-methyl­sulfonyl-1,2,3,6,7,7a-hexa­hydro-3a,6-ep­oxy­iso­indole (ERIVIL; Temel *et al.*, 2011 ▸) and *tert*-butyl 3a-chloro­per-hydro-2,6a-ep­oxy­oxireno(*e*)isoindole-5-carboxyl­ate (MIGTIG; Koşar *et al.*, 2007 ▸).

In the crystal of IMUBIE, the mol­ecules are linked into dimers by pairs of C—H⋯O hydrogen bonds, thus generating 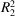
(18) rings. The crystal packing is dominated by H⋯H, Br⋯H, H⋯π and Br⋯π inter­actions. In the crystal structures of OMEMAX, AGONUH, TIJMIK, YAXCIL, UPAQEI and ERIVIL, the mol­ecules are linked by predominantly C—H⋯O hydrogen bonds describing different hydrogen-bonding pattern connectivities. In OMEMAX, mol­ecules form sheets lying parallel to the (002) plane. These sheets are connected only by weak van der Waals inter­actions. In the crystal of AGONUH, the mol­ecules are connected in zigzag chains running along the *b*-axis direction. In TIJMIK, two types of C—H⋯O hydrogen bonds are found, *viz. R* ^2^_2_(20) and 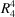
(26) rings, with adjacent rings running parallel to the *ac* plane. Additionally, C—H⋯O hydrogen bonds form a *C*(6) chain, linking the mol­ecules in the *b*-axis direction. In the crystal of ERIVIL, the mol­ecules are connected into 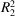
(8) and 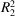
(14) rings along the *b*-axis direction. In MIGTIG, the mol­ecules are linked only by weak van der Waals inter­actions.

## Synthesis and crystallization   

(3a*S*,6*S*,7a*R*)-2-(4-(Tri­fluoro­meth­yl)phen­yl)-2,3,7,7a-tetra­hydro-3a,6-ep­oxy­isoindol-1(6*H*)-one (1.2 mmol) and the brominating agent [(Me_2_NCOMe)_2_H]Br_3_ (1.32 mmol) in 3 mL of dry chloro­form were heated under reflux for 3–5 h (TLC control, EtOAc–hexane, 1:1). The reaction mixture was poured into H_2_O (50 mL), extracted with CHCl_3_ (3 × 20 mL) and combined organic parts were dried over anhydrous Na_2_SO_4_ and the solvent was evaporated under reduced pressure. Recrystallization of the obtained residue from a hexa­ne–AcOEt mixture gave single crystals suitable for X-ray analysis.

Yield 15%, m.p. > 438 K (decomp.), pale-beige plates. ^1^H NMR (600.2 MHz, CDCl_3_) δ 7.79 (*d*, *J =* 8.1 Hz, 2H, H-3, H-5 H_arom._), 7.64 (*d*, *J* = 8.1 Hz, 2H, H-2, H-6 H_arom._), 4.78 (*t*, *J* = 5.0 Hz, 1H, H-6), 4.53 (*ddd*, *J* = 4.0 Hz, *J* = 1.3 Hz, *J* = 2.3 Hz, 1H, H-5), 4.26 (*t*, *J* = 2.3 Hz, 1H, H-4), 4.13 (*dd*, *J* = 11.6 Hz, *J* = 1.7 Hz, 1H), 4.09 (*dd*, *J* = 11.6 Hz, *J* = 1.7 Hz, 1H, H-3), 2.99 (*dd*, *J* = 4.5 Hz, *J =* 9.1 Hz, 1H, H-7a), 2.83 (*ddd*, *J* = 1.7 Hz, *J* = 9.1 Hz, *J* = 13.1 Hz, 1H, H-7B), 2.35–2.31 (*m*, 1H, H-7A). ^13^C NMR (150.9 MHz, CDCl_3_) δ 172.7, 141.6, 126.7 (*q*, *J =* 33.2 Hz, 1C), 126.2 (*q*, *J* = 2.9 Hz, 2C), 123.8 (*J =* 271.6 Hz, 1C), 119.4 (2C), 88.7, 80.1, 55.5, 53.8, 50.5, 49.7, 30.9. ^19^F NMR (564.7 MHz, CDCl_3_) δ −62.1. IR (KBr): 1703 (NC=O). MS (ESI): *m*/*z* = 456 [*M* + H^+^]. Analysis calculated for C_15_H_12_Br_2_F_3_NO_2_: C 39.59%, H 2.66%, N 3.08%. Found: C 39.55%, H 2.61%, N 3.20%.

## Refinement   

Crystal data, data collection and structure refinement details are summarized in Table 4 ▸. All C-bound H atoms were placed at calculated positions using a riding model, with aromatic C—H = 0.93–0.98 Å, and with *U*_iso_(H) = 1.2*U*_eq_(C). The F atoms of the tri­fluoro­methyl groups (CF_3_) of both mol­ecules are disordered over two sets of sites with refined site occupancies of 0.60 (3)/0.40 (3) for mol­ecule *A* and 0.640 (15)/0.360 (15) for mol­ecule *B*. The major and minor components of the disordered CF_3_ groups of mol­ecules *A* and *B* were restrained to have approximately equal C—F distances by use of the *SHELXL* SADI instruction. The anisotropies of the F1, F2, F3, F1*A*, F2*A*, F3*A*, F21, F22, F23, F21*A*, F22*A* and F23*A* atoms were restrained with ISOR 0.01 0.02 in *SHELXL* (Sheldrick, 2015*b* ▸). Six outliers (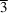 1 4, 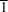 2 11, 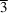 2 5, 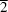 7 7, 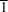 4 7 and 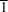 10 25) were omitted in the final refinement.

## Supplementary Material

Crystal structure: contains datablock(s) I. DOI: 10.1107/S2056989021003200/zn2006sup1.cif

Structure factors: contains datablock(s) I. DOI: 10.1107/S2056989021003200/zn2006Isup2.hkl

Supporting information file. DOI: 10.1107/S2056989021003200/zn2006Isup3.cml

CCDC reference: 2036842

Additional supporting information:  crystallographic information; 3D view; checkCIF report

## Figures and Tables

**Table 1:** Hydrogen-bond geometry (Å, °) *Cg*5 and *Cg*10 are the centroids of the C11–C16 and C31–C36 rings, respectively.

*D*—H⋯*A*	*D*—H	H⋯*A*	*D*⋯*A*	*D*—H⋯*A*
C5—H5*A*⋯O8^i^	0.98	2.57	3.342 (4)	135
C7—H7*A*⋯Br2	0.97	2.82	3.300 (4)	112
C16—H16*A*⋯O1	0.93	2.26	2.853 (4)	121
C27—H27*A*⋯Br22	0.97	2.78	3.259 (4)	111
C36—H36*A*⋯O21	0.93	2.28	2.856 (5)	120
C7*A*—H7*AA*⋯*Cg*10^i^	0.98	2.94	3.741 (4)	139
C27*A*—H27*C*⋯*Cg*5^i^	0.98	2.97	3.924 (4)	166

Symmetry code: (i) -x+1, -y+1, -z+1.

**Table 2:** Summary of short inter­atomic contacts (Å) in the title compound Asterisks indicate symmetry-generated atoms

Contact	Distance	Symmetry operation
H5*A*⋯O8	2.57	1 − *x*, 1 − *y*, 1 − *z*
H3*B*⋯O28	2.82	*x*, {3\over 2} − *y*, − {1\over 2} + *z*
H4*A*⋯O1	2.74	−1 + *x*, *y*, *z*
Br2⋯Br22	3.74	−*x*, −{1\over 2} + *y*, {3\over 2} − *z*
Br2⋯H26*A*	2.95	1 − *x*, −{1\over 2} + *y*, {3\over 2} − *z*
H7*AA*⋯C36	2.59	1 − *x*, 1 − *y*, 1 − *z*
*F3*A*⋯*F23	2.90	2 − *x*, −{1\over 2} + *y*, {1\over 2} − *z*
H15*A*⋯O21	2.62	2 − *x*, 1 − *y*, 1 − *z*
*F2*A*⋯H23*B*	2.60	1 + *x*, {3\over 2} − *y*, −{1\over 2} + *z*
H23*A*⋯H36*A*	2.49	−1 + *x*, *y*, *z*
H23*B*⋯*F2*A*	2.60	−1 + *x*, {3\over 2} − *y*, {1\over 2} + *z*
Br22⋯*F23	3.48	−1 + *x*, {3\over 2} − *y*, {1\over 2} + *z*
*F22*A*⋯*F1	2.94	2 − *x*, {1\over 2} + *y*, {1\over 2} − *z*
H33*A*⋯*F21*A*	2.84	1 − *x*, 2 − *y*, 1 − *z*
*F22*A*⋯*F22	3.09	2 − *x*, 2 − *y*, 1 − *z*

**Table 3:** Percentage contributions of inter­atomic contacts to the Hirshfeld surface for the mol­ecules *A* and *B* of the title compound

Contact	Mol­ecule *A*	Mol­ecule *B*
H⋯H	23.8	22.4
Br⋯H/H⋯Br	18.3	12.3
O⋯H/H⋯O	14.3	9.7
F⋯H/H⋯F	10.4	19.1
C⋯H/H⋯C	9.9	7.8
F⋯F	6.9	8.6
Br⋯F/F⋯Br	3.9	8.0
Br⋯C/C⋯Br	3.7	3.5
Br⋯Br	2.4	1.6
F⋯C/C⋯F	2.3	2.4
Br⋯O/O⋯Br	1.4	2.1
Br⋯N/N⋯Br	1.1	0.9
O⋯N/N⋯O	0.5	0.5
O⋯C/C⋯O	0.5	0.4
C⋯C	0.3	0.3
N⋯H/H⋯N	0.2	0.3
N⋯C/C⋯N	0.1	0.1

**Table 4:** Experimental details

Crystal data	
Chemical formula	C_15_H_12_Br_2_F_3_NO_2_
*M*_r_	455.08
Crystal system, space group	Monoclinic, *P*2_1_/*c*
Temperature (K)	296
*a*, *b*, *c* (Å)	6.6543 (2), 18.9031 (5), 25.1995 (7)
β (°)	97.132 (2)
*V* (Å^3^)	3145.24 (15)
*Z*	8
Radiation type	Mo *K*α
μ (mm^−1^)	5.19
Crystal size (mm)	0.44 × 0.12 × 0.04

Data collection	
Diffractometer	Bruker *KAPPA* APEXII area-detector
Absorption correction	Multi-scan (*SADABS*; Bruker, 2013 ▸)
*T*_min_, *T*_max_	0.704, 0.819
No. of measured, independent and observed [*I* > 2σ(*I*)] reflections	44884, 7199, 4255
*R*_int_	0.057
(sin θ/λ)_max_ (Å^−1^)	0.650

Refinement	
*R*[*F* ^2^ > 2σ(*F* ^2^)], *wR*(*F* ^2^), *S*	0.038, 0.077, 1.00
No. of reflections	7199
No. of parameters	471
No. of restraints	204
H-atom treatment	H-atom parameters constrained
Δρ_max_, Δρ_min_ (e Å^−3^)	0.52, −0.44

Computer programs: *APEX2* and *SAINT* (Bruker, 2013 ▸), *SHELXT* (Sheldrick, 2015*a* ▸), *SHELXL* (Sheldrick, 2015*b* ▸), *ORTEP-3 for Windows* (Farrugia, 2012 ▸) and *PLATON* (Spek, 2020 ▸).

## supplementary crystallographic information

### Crystal data

**Table:**

C_15_H_12_Br_2_F_3_NO_2_	*F*(000) = 1776
*M**_r_* = 455.08	*D*_x_ = 1.922 Mg m^−^^3^
Monoclinic, *P*2_1_/*c*	Mo *K*α radiation, λ = 0.71073 Å
*a* = 6.6543 (2) Å	Cell parameters from 6983 reflections
*b* = 18.9031 (5) Å	θ = 3.1–22.1°
*c* = 25.1995 (7) Å	µ = 5.19 mm^−^^1^
β = 97.132 (2)°	*T* = 296 K
*V* = 3145.24 (15) Å^3^	Plate, light beige
*Z* = 8	0.44 × 0.12 × 0.04 mm

### Data collection

**Table:**

Bruker KAPPA APEXII area-detector diffractometer	4255 reflections with *I* > 2σ(*I*)
φ and ω scans	*R*_int_ = 0.057
Absorption correction: multi-scan (SADABS; Bruker, 2013)	θ_max_ = 27.5°, θ_min_ = 4.2°
*T*_min_ = 0.704, *T*_max_ = 0.819	*h* = −8→8
44884 measured reflections	*k* = −24→24
7199 independent reflections	*l* = −32→32

### Refinement

**Table:**

Refinement on *F*^2^	204 restraints
Least-squares matrix: full	Hydrogen site location: inferred from neighbouring sites
*R*[*F*^2^ > 2σ(*F*^2^)] = 0.038	H-atom parameters constrained
*wR*(*F*^2^) = 0.077	*w* = 1/[σ^2^(*F*_o_^2^) + (0.0254*P*)^2^ + 2.067*P*] where *P* = (*F*_o_^2^ + 2*F*_c_^2^)/3
*S* = 1.00	(Δ/σ)_max_ = 0.001
7199 reflections	Δρ_max_ = 0.52 e Å^−^^3^
471 parameters	Δρ_min_ = −0.44 e Å^−^^3^

### Special details

**Table:**

Geometry. All esds (except the esd in the dihedral angle between two l.s. planes) are estimated using the full covariance matrix. The cell esds are taken into account individually in the estimation of esds in distances, angles and torsion angles; correlations between esds in cell parameters are only used when they are defined by crystal symmetry. An approximate (isotropic) treatment of cell esds is used for estimating esds involving l.s. planes.

### Fractional atomic coordinates and isotropic or equivalent isotropic displacement parameters (Å^2^)

**Table:**

	*x*	*y*	*z*	*U*_iso_*/*U*_eq_	Occ. (<1)
Br1	0.04969 (6)	0.49524 (2)	0.40882 (2)	0.05936 (12)	
Br2	0.16433 (6)	0.33168 (2)	0.52432 (2)	0.05972 (13)	
F1	0.8250 (14)	0.4392 (8)	0.1004 (3)	0.100 (3)	0.60 (3)
F2	0.984 (3)	0.5243 (3)	0.1380 (4)	0.097 (3)	0.60 (3)
F3	1.1221 (17)	0.4233 (8)	0.1395 (5)	0.105 (4)	0.60 (3)
F1A	0.8229 (18)	0.4756 (13)	0.1027 (6)	0.099 (5)	0.40 (3)
F2A	1.084 (3)	0.5104 (9)	0.1523 (7)	0.100 (5)	0.40 (3)
F3A	1.056 (3)	0.4043 (6)	0.1284 (6)	0.082 (4)	0.40 (3)
O1	0.7970 (4)	0.29869 (13)	0.36728 (11)	0.0602 (7)	
O8	0.5397 (3)	0.44953 (12)	0.44391 (9)	0.0477 (6)	
N2	0.6066 (4)	0.39552 (14)	0.33412 (11)	0.0394 (7)	
C1	0.6561 (5)	0.33992 (18)	0.36860 (14)	0.0423 (8)	
C3	0.4324 (5)	0.43625 (18)	0.34806 (13)	0.0417 (8)	
H3A	0.311584	0.426135	0.323570	0.050*	
H3B	0.459315	0.486670	0.347699	0.050*	
C3A	0.4104 (5)	0.41102 (17)	0.40351 (13)	0.0368 (8)	
C4	0.2098 (5)	0.41007 (17)	0.42722 (13)	0.0394 (8)	
H4A	0.131785	0.367487	0.416059	0.047*	
C5	0.2862 (5)	0.40741 (18)	0.48724 (13)	0.0436 (8)	
H5A	0.254387	0.452572	0.503380	0.052*	
C6	0.5167 (5)	0.4022 (2)	0.48785 (14)	0.0489 (9)	
H6A	0.592991	0.417081	0.521802	0.059*	
C7	0.5857 (5)	0.3323 (2)	0.46770 (15)	0.0548 (10)	
H7A	0.526303	0.292812	0.484771	0.066*	
H7B	0.732082	0.328072	0.473085	0.066*	
C7A	0.5045 (5)	0.33729 (17)	0.40819 (13)	0.0387 (8)	
H7AA	0.402046	0.300829	0.398258	0.046*	
C11	0.6955 (5)	0.41029 (17)	0.28750 (14)	0.0399 (8)	
C12	0.5936 (6)	0.4528 (2)	0.24827 (16)	0.0553 (10)	
H12A	0.468838	0.472055	0.253387	0.066*	
C13	0.6754 (6)	0.4671 (2)	0.20159 (16)	0.0613 (11)	
H13A	0.604994	0.495703	0.175597	0.074*	
C14	0.8598 (6)	0.43931 (19)	0.19327 (15)	0.0498 (9)	
C15	0.9620 (5)	0.39722 (19)	0.23196 (15)	0.0486 (9)	
H15A	1.087012	0.378300	0.226660	0.058*	
C16	0.8817 (5)	0.38265 (18)	0.27862 (14)	0.0468 (9)	
H16A	0.952961	0.353997	0.304433	0.056*	
C17	0.9517 (7)	0.4566 (2)	0.14398 (18)	0.0672 (12)	
Br21	−0.02652 (5)	0.90131 (2)	0.77414 (2)	0.05796 (12)	
Br22	0.19224 (7)	0.74494 (2)	0.89213 (2)	0.07068 (14)	
F21	0.6437 (15)	0.8625 (7)	0.4491 (3)	0.138 (4)	0.640 (15)
F22	0.7980 (19)	0.9531 (3)	0.4818 (4)	0.113 (3)	0.640 (15)
F23	0.9516 (12)	0.8560 (6)	0.4812 (3)	0.116 (3)	0.640 (15)
F21A	0.674 (3)	0.9420 (7)	0.4653 (6)	0.111 (5)	0.360 (15)
F22A	0.9709 (17)	0.9064 (12)	0.4937 (5)	0.137 (6)	0.360 (15)
F23A	0.751 (3)	0.8377 (5)	0.4539 (4)	0.094 (4)	0.360 (15)
O21	0.7477 (4)	0.73028 (14)	0.72019 (11)	0.0656 (8)	
O28	0.4682 (3)	0.87970 (12)	0.79846 (9)	0.0481 (6)	
N22	0.5250 (4)	0.81952 (15)	0.69035 (11)	0.0414 (7)	
C21	0.6025 (5)	0.76761 (19)	0.72515 (15)	0.0451 (9)	
C23	0.3412 (5)	0.85182 (19)	0.70647 (13)	0.0422 (8)	
H23A	0.220656	0.833540	0.685316	0.051*	
H23B	0.344086	0.902874	0.702787	0.051*	
C23A	0.3480 (4)	0.83071 (17)	0.76374 (13)	0.0372 (8)	
C24	0.1603 (5)	0.82315 (18)	0.79201 (14)	0.0425 (8)	
H24A	0.093727	0.777509	0.783839	0.051*	
C25	0.2563 (5)	0.82648 (19)	0.85095 (14)	0.0481 (9)	
H25A	0.207680	0.869136	0.867414	0.058*	
C26	0.4823 (5)	0.8359 (2)	0.84539 (15)	0.0538 (10)	
H26A	0.558598	0.858168	0.876756	0.065*	
C27	0.5823 (5)	0.7695 (2)	0.82783 (15)	0.0578 (11)	
H27A	0.555775	0.729042	0.849561	0.069*	
H27B	0.727365	0.775539	0.828410	0.069*	
C27A	0.4739 (5)	0.76308 (18)	0.77028 (14)	0.0431 (8)	
H27C	0.386791	0.721131	0.766510	0.052*	
C31	0.5911 (5)	0.83652 (18)	0.64070 (14)	0.0418 (8)	
C32	0.4639 (6)	0.8728 (2)	0.60261 (15)	0.0556 (10)	
H32A	0.336348	0.886497	0.610202	0.067*	
C33	0.5228 (7)	0.8889 (2)	0.55368 (17)	0.0674 (12)	
H33A	0.435143	0.913322	0.528468	0.081*	
C34	0.7117 (7)	0.8691 (2)	0.54186 (17)	0.0624 (11)	
C35	0.8395 (6)	0.8327 (2)	0.57932 (17)	0.0597 (11)	
H35A	0.966193	0.818608	0.571272	0.072*	
C36	0.7822 (5)	0.81668 (19)	0.62877 (16)	0.0508 (9)	
H36A	0.870853	0.792753	0.654043	0.061*	
C37	0.7765 (9)	0.8866 (3)	0.4889 (2)	0.0852 (15)	

### Atomic displacement parameters (Å^2^)

**Table:**

	*U*^11^	*U*^22^	*U*^33^	*U*^12^	*U*^13^	*U*^23^
Br1	0.0517 (2)	0.0598 (3)	0.0702 (3)	0.01235 (19)	0.0221 (2)	0.0094 (2)
Br2	0.0562 (2)	0.0703 (3)	0.0529 (3)	−0.0180 (2)	0.00743 (19)	0.0129 (2)
F1	0.139 (5)	0.110 (7)	0.053 (4)	−0.014 (4)	0.028 (3)	0.003 (4)
F2	0.146 (7)	0.066 (4)	0.090 (5)	−0.004 (4)	0.059 (5)	0.013 (3)
F3	0.098 (5)	0.135 (7)	0.095 (6)	0.033 (5)	0.060 (4)	0.032 (5)
F1A	0.106 (6)	0.124 (9)	0.073 (6)	0.007 (6)	0.033 (5)	0.039 (6)
F2A	0.111 (8)	0.104 (8)	0.093 (7)	−0.056 (6)	0.048 (6)	−0.011 (5)
F3A	0.123 (8)	0.063 (5)	0.075 (6)	0.000 (5)	0.065 (6)	−0.016 (4)
O1	0.0612 (16)	0.0520 (16)	0.0717 (19)	0.0192 (13)	0.0251 (14)	0.0135 (14)
O8	0.0473 (14)	0.0514 (15)	0.0459 (15)	−0.0230 (11)	0.0111 (11)	−0.0137 (12)
N2	0.0396 (15)	0.0386 (16)	0.0414 (17)	0.0037 (13)	0.0107 (13)	−0.0023 (14)
C1	0.0430 (19)	0.038 (2)	0.046 (2)	−0.0013 (16)	0.0085 (17)	−0.0035 (17)
C3	0.0440 (19)	0.038 (2)	0.045 (2)	0.0012 (15)	0.0123 (16)	−0.0014 (16)
C3A	0.0372 (17)	0.038 (2)	0.0360 (19)	−0.0049 (15)	0.0076 (15)	−0.0069 (15)
C4	0.0397 (18)	0.0359 (19)	0.043 (2)	−0.0047 (15)	0.0074 (16)	−0.0032 (16)
C5	0.047 (2)	0.044 (2)	0.041 (2)	−0.0137 (16)	0.0122 (16)	−0.0071 (17)
C6	0.043 (2)	0.069 (3)	0.034 (2)	−0.0185 (19)	−0.0001 (16)	−0.0061 (19)
C7	0.042 (2)	0.071 (3)	0.051 (2)	0.0065 (19)	0.0047 (18)	0.010 (2)
C7A	0.0369 (18)	0.0373 (19)	0.042 (2)	−0.0022 (15)	0.0069 (15)	−0.0033 (16)
C11	0.0433 (19)	0.0356 (19)	0.042 (2)	−0.0004 (16)	0.0106 (16)	−0.0053 (16)
C12	0.056 (2)	0.054 (2)	0.060 (3)	0.0161 (19)	0.023 (2)	0.010 (2)
C13	0.073 (3)	0.058 (3)	0.056 (3)	0.022 (2)	0.021 (2)	0.014 (2)
C14	0.062 (2)	0.042 (2)	0.049 (2)	0.0024 (18)	0.022 (2)	−0.0051 (18)
C15	0.046 (2)	0.054 (2)	0.049 (2)	0.0034 (18)	0.0199 (18)	−0.0035 (19)
C16	0.048 (2)	0.046 (2)	0.046 (2)	0.0053 (17)	0.0057 (17)	−0.0022 (18)
C17	0.085 (3)	0.061 (3)	0.060 (3)	0.011 (3)	0.029 (3)	−0.001 (3)
Br21	0.0449 (2)	0.0658 (3)	0.0640 (3)	0.01529 (19)	0.01001 (18)	−0.0037 (2)
Br22	0.0775 (3)	0.0783 (3)	0.0609 (3)	0.0063 (2)	0.0271 (2)	0.0142 (2)
F21	0.152 (6)	0.201 (8)	0.066 (4)	−0.033 (5)	0.033 (4)	−0.014 (5)
F22	0.168 (7)	0.079 (4)	0.105 (5)	−0.015 (4)	0.070 (5)	0.016 (3)
F23	0.133 (5)	0.135 (6)	0.098 (4)	0.027 (4)	0.084 (4)	0.020 (4)
F21A	0.140 (9)	0.106 (8)	0.096 (7)	0.033 (6)	0.058 (6)	0.041 (6)
F22A	0.127 (8)	0.169 (11)	0.126 (8)	−0.033 (7)	0.058 (6)	0.029 (7)
F23A	0.155 (9)	0.073 (6)	0.060 (5)	0.006 (6)	0.044 (6)	−0.004 (4)
O21	0.0596 (16)	0.0704 (19)	0.071 (2)	0.0285 (14)	0.0227 (14)	0.0036 (15)
O28	0.0453 (13)	0.0506 (15)	0.0469 (15)	−0.0105 (11)	−0.0005 (11)	−0.0058 (12)
N22	0.0339 (14)	0.0492 (18)	0.0421 (17)	0.0020 (13)	0.0088 (13)	−0.0051 (14)
C21	0.0384 (19)	0.047 (2)	0.050 (2)	0.0017 (17)	0.0064 (17)	−0.0056 (18)
C23	0.0351 (17)	0.049 (2)	0.043 (2)	0.0041 (15)	0.0061 (15)	−0.0030 (17)
C23A	0.0323 (16)	0.0379 (19)	0.041 (2)	−0.0039 (15)	0.0022 (15)	−0.0074 (16)
C24	0.0396 (18)	0.042 (2)	0.047 (2)	0.0026 (15)	0.0097 (16)	−0.0066 (17)
C25	0.054 (2)	0.050 (2)	0.043 (2)	0.0045 (18)	0.0140 (17)	−0.0065 (18)
C26	0.051 (2)	0.068 (3)	0.040 (2)	−0.005 (2)	−0.0041 (18)	−0.006 (2)
C27	0.042 (2)	0.079 (3)	0.052 (3)	0.013 (2)	0.0056 (18)	0.009 (2)
C27A	0.0360 (18)	0.042 (2)	0.052 (2)	0.0030 (15)	0.0069 (16)	0.0021 (17)
C31	0.0422 (19)	0.044 (2)	0.040 (2)	−0.0074 (16)	0.0101 (16)	−0.0095 (17)
C32	0.053 (2)	0.066 (3)	0.050 (3)	0.009 (2)	0.015 (2)	0.001 (2)
C33	0.076 (3)	0.069 (3)	0.059 (3)	0.005 (2)	0.019 (2)	0.007 (2)
C34	0.081 (3)	0.055 (3)	0.056 (3)	−0.015 (2)	0.028 (2)	−0.010 (2)
C35	0.057 (2)	0.059 (3)	0.068 (3)	−0.012 (2)	0.028 (2)	−0.017 (2)
C36	0.042 (2)	0.057 (2)	0.055 (2)	−0.0074 (17)	0.0092 (18)	−0.0097 (19)
C37	0.113 (5)	0.080 (4)	0.069 (4)	−0.019 (4)	0.038 (3)	−0.003 (3)

### Geometric parameters (Å, º)

**Table:**

Br1—C4	1.954 (3)	Br21—C24	1.947 (3)
Br2—C5	1.941 (3)	Br22—C25	1.935 (4)
F1—F1A	0.691 (15)	F21—F23A	0.852 (11)
F1—C17	1.341 (7)	F21—C37	1.332 (7)
F1—F3A	1.738 (15)	F21—F21A	1.564 (13)
F2—F2A	0.764 (14)	F22—F21A	0.899 (13)
F2—C17	1.308 (7)	F22—C37	1.280 (7)
F2—F1A	1.596 (14)	F22—F22A	1.452 (14)
F3—F3A	0.610 (16)	F23—F22A	1.007 (15)
F3—C17	1.314 (7)	F23—C37	1.337 (7)
F3—F2A	1.703 (13)	F23—F23A	1.463 (13)
F1A—C17	1.313 (9)	F21A—C37	1.347 (9)
F2A—C17	1.345 (9)	F22A—C37	1.338 (9)
F3A—C17	1.295 (9)	F23A—C37	1.275 (9)
O1—C1	1.223 (4)	O21—C21	1.215 (4)
O8—C3A	1.446 (4)	O28—C26	1.437 (4)
O8—C6	1.446 (4)	O28—C23A	1.445 (4)
N2—C1	1.377 (4)	N22—C21	1.373 (4)
N2—C11	1.407 (4)	N22—C31	1.414 (4)
N2—C3	1.470 (4)	N22—C23	1.469 (4)
C1—C7A	1.505 (4)	C21—C27A	1.508 (5)
C3—C3A	1.501 (4)	C23—C23A	1.492 (5)
C3—H3A	0.9700	C23—H23A	0.9700
C3—H3B	0.9700	C23—H23B	0.9700
C3A—C7A	1.527 (4)	C23A—C24	1.520 (4)
C3A—C4	1.529 (4)	C23A—C27A	1.526 (4)
C4—C5	1.535 (5)	C24—C25	1.543 (5)
C4—H4A	0.9800	C24—H24A	0.9800
C5—C6	1.536 (5)	C25—C26	1.538 (5)
C5—H5A	0.9800	C25—H25A	0.9800
C6—C7	1.508 (5)	C26—C27	1.511 (5)
C6—H6A	0.9800	C26—H26A	0.9800
C7—C7A	1.532 (5)	C27—C27A	1.542 (5)
C7—H7A	0.9700	C27—H27A	0.9700
C7—H7B	0.9700	C27—H27B	0.9700
C7A—H7AA	0.9800	C27A—H27C	0.9800
C11—C12	1.385 (5)	C31—C32	1.380 (5)
C11—C16	1.388 (4)	C31—C36	1.394 (5)
C12—C13	1.382 (5)	C32—C33	1.374 (5)
C12—H12A	0.9300	C32—H32A	0.9300
C13—C14	1.375 (5)	C33—C34	1.379 (6)
C13—H13A	0.9300	C33—H33A	0.9300
C14—C15	1.372 (5)	C34—C35	1.374 (6)
C14—C17	1.488 (5)	C34—C37	1.489 (6)
C15—C16	1.379 (5)	C35—C36	1.381 (5)
C15—H15A	0.9300	C35—H35A	0.9300
C16—H16A	0.9300	C36—H36A	0.9300
			
F1A—F1—C17	72.7 (11)	F23A—F21—C37	67.4 (8)
F1A—F1—F3A	111.7 (13)	F23A—F21—F21A	114.4 (10)
C17—F1—F3A	47.6 (5)	C37—F21—F21A	54.7 (5)
F2A—F2—C17	76.0 (9)	F21A—F22—C37	74.0 (8)
F2A—F2—F1A	123.5 (11)	F21A—F22—F22A	127.7 (11)
C17—F2—F1A	52.6 (4)	C37—F22—F22A	58.3 (5)
F3A—F3—C17	74.8 (13)	F22A—F23—C37	68.0 (7)
F3A—F3—F2A	122.7 (14)	F22A—F23—F23A	116.4 (9)
C17—F3—F2A	51.0 (4)	C37—F23—F23A	54.0 (4)
F1—F1A—C17	77.2 (11)	F22—F21A—C37	66.0 (7)
F1—F1A—F2	127.1 (13)	F22—F21A—F21	115.1 (10)
C17—F1A—F2	52.4 (5)	C37—F21A—F21	53.8 (5)
F2—F2A—C17	70.6 (9)	F23—F22A—C37	67.8 (7)
F2—F2A—F3	112.5 (12)	F23—F22A—F22	116.3 (9)
C17—F2A—F3	49.4 (4)	C37—F22A—F22	54.5 (5)
F3—F3A—C17	78.2 (12)	F21—F23A—C37	74.6 (8)
F3—F3A—F1	121.1 (15)	F21—F23A—F23	129.5 (10)
C17—F3A—F1	49.9 (5)	C37—F23A—F23	58.0 (5)
C3A—O8—C6	96.7 (2)	C26—O28—C23A	96.0 (2)
C1—N2—C11	125.8 (3)	C21—N22—C31	126.4 (3)
C1—N2—C3	112.5 (3)	C21—N22—C23	112.3 (3)
C11—N2—C3	121.4 (3)	C31—N22—C23	120.8 (3)
O1—C1—N2	126.4 (3)	O21—C21—N22	126.1 (3)
O1—C1—C7A	125.2 (3)	O21—C21—C27A	125.3 (3)
N2—C1—C7A	108.4 (3)	N22—C21—C27A	108.6 (3)
N2—C3—C3A	103.1 (3)	N22—C23—C23A	103.3 (3)
N2—C3—H3A	111.1	N22—C23—H23A	111.1
C3A—C3—H3A	111.1	C23A—C23—H23A	111.1
N2—C3—H3B	111.1	N22—C23—H23B	111.1
C3A—C3—H3B	111.1	C23A—C23—H23B	111.1
H3A—C3—H3B	109.1	H23A—C23—H23B	109.1
O8—C3A—C3	112.2 (3)	O28—C23A—C23	111.2 (3)
O8—C3A—C7A	101.7 (2)	O28—C23A—C24	101.6 (3)
C3—C3A—C7A	106.0 (3)	C23—C23A—C24	123.4 (3)
O8—C3A—C4	101.7 (2)	O28—C23A—C27A	102.5 (2)
C3—C3A—C4	124.0 (3)	C23—C23A—C27A	106.2 (3)
C7A—C3A—C4	109.2 (3)	C24—C23A—C27A	110.0 (3)
C3A—C4—C5	100.8 (2)	C23A—C24—C25	100.4 (3)
C3A—C4—Br1	112.0 (2)	C23A—C24—Br21	111.0 (2)
C5—C4—Br1	111.4 (2)	C25—C24—Br21	111.6 (2)
C3A—C4—H4A	110.8	C23A—C24—H24A	111.1
C5—C4—H4A	110.8	C25—C24—H24A	111.1
Br1—C4—H4A	110.8	Br21—C24—H24A	111.1
C4—C5—C6	102.7 (3)	C26—C25—C24	102.1 (3)
C4—C5—Br2	113.0 (2)	C26—C25—Br22	115.1 (3)
C6—C5—Br2	115.1 (2)	C24—C25—Br22	113.0 (2)
C4—C5—H5A	108.6	C26—C25—H25A	108.8
C6—C5—H5A	108.6	C24—C25—H25A	108.8
Br2—C5—H5A	108.6	Br22—C25—H25A	108.8
O8—C6—C7	102.6 (3)	O28—C26—C27	102.9 (3)
O8—C6—C5	98.8 (3)	O28—C26—C25	100.2 (3)
C7—C6—C5	113.5 (3)	C27—C26—C25	113.7 (3)
O8—C6—H6A	113.5	O28—C26—H26A	113.0
C7—C6—H6A	113.5	C27—C26—H26A	113.0
C5—C6—H6A	113.5	C25—C26—H26A	113.0
C6—C7—C7A	101.0 (3)	C26—C27—C27A	99.7 (3)
C6—C7—H7A	111.6	C26—C27—H27A	111.8
C7A—C7—H7A	111.6	C27A—C27—H27A	111.8
C6—C7—H7B	111.6	C26—C27—H27B	111.8
C7A—C7—H7B	111.6	C27A—C27—H27B	111.8
H7A—C7—H7B	109.4	H27A—C27—H27B	109.5
C1—C7A—C3A	102.8 (3)	C21—C27A—C23A	103.1 (3)
C1—C7A—C7	117.8 (3)	C21—C27A—C27	117.5 (3)
C3A—C7A—C7	102.9 (3)	C23A—C27A—C27	102.8 (3)
C1—C7A—H7AA	110.9	C21—C27A—H27C	110.9
C3A—C7A—H7AA	110.9	C23A—C27A—H27C	110.9
C7—C7A—H7AA	110.9	C27—C27A—H27C	110.9
C12—C11—C16	118.1 (3)	C32—C31—C36	118.7 (3)
C12—C11—N2	119.5 (3)	C32—C31—N22	119.8 (3)
C16—C11—N2	122.4 (3)	C36—C31—N22	121.5 (3)
C13—C12—C11	120.8 (3)	C33—C32—C31	121.0 (4)
C13—C12—H12A	119.6	C33—C32—H32A	119.5
C11—C12—H12A	119.6	C31—C32—H32A	119.5
C14—C13—C12	120.6 (4)	C32—C33—C34	120.2 (4)
C14—C13—H13A	119.7	C32—C33—H33A	119.9
C12—C13—H13A	119.7	C34—C33—H33A	119.9
C15—C14—C13	119.1 (3)	C35—C34—C33	119.4 (4)
C15—C14—C17	120.2 (3)	C35—C34—C37	120.2 (4)
C13—C14—C17	120.7 (4)	C33—C34—C37	120.4 (5)
C14—C15—C16	120.8 (3)	C34—C35—C36	120.8 (4)
C14—C15—H15A	119.6	C34—C35—H35A	119.6
C16—C15—H15A	119.6	C36—C35—H35A	119.6
C15—C16—C11	120.7 (3)	C35—C36—C31	119.8 (4)
C15—C16—H16A	119.6	C35—C36—H36A	120.1
C11—C16—H16A	119.6	C31—C36—H36A	120.1
F3A—C17—F2	127.6 (8)	F23A—C37—F22	128.3 (7)
F3A—C17—F1A	106.8 (9)	F22—C37—F21	107.7 (7)
F2—C17—F1A	75.0 (7)	F23A—C37—F23	68.1 (6)
F2—C17—F3	107.6 (6)	F22—C37—F23	106.8 (6)
F1A—C17—F3	123.2 (9)	F21—C37—F23	104.5 (6)
F3A—C17—F1	82.5 (8)	F23A—C37—F22A	107.8 (8)
F2—C17—F1	103.9 (6)	F22—C37—F22A	67.3 (7)
F3—C17—F1	105.9 (6)	F21—C37—F22A	134.9 (8)
F3A—C17—F2A	105.1 (7)	F23A—C37—F21A	104.0 (8)
F1A—C17—F2A	105.5 (7)	F21—C37—F21A	71.4 (7)
F3—C17—F2A	79.7 (7)	F23—C37—F21A	132.3 (7)
F1—C17—F2A	130.3 (8)	F22A—C37—F21A	104.6 (8)
F3A—C17—C14	112.0 (7)	F23A—C37—C34	115.6 (6)
F2—C17—C14	113.7 (5)	F22—C37—C34	113.3 (5)
F1A—C17—C14	115.2 (7)	F21—C37—C34	111.1 (5)
F3—C17—C14	114.5 (6)	F23—C37—C34	112.9 (5)
F1—C17—C14	110.4 (5)	F22A—C37—C34	111.6 (7)
F2A—C17—C14	111.5 (7)	F21A—C37—C34	112.5 (7)
			
F3A—F1—F1A—C17	−27.9 (13)	F22A—F22—F21A—C37	24.2 (13)
C17—F1—F1A—F2	16.9 (19)	C37—F22—F21A—F21	−22.7 (10)
F3A—F1—F1A—F2	−11 (3)	F22A—F22—F21A—F21	1 (2)
F2A—F2—F1A—F1	8 (4)	F23A—F21—F21A—F22	−7 (2)
C17—F2—F1A—F1	−21 (2)	C37—F21—F21A—F22	25.9 (12)
F2A—F2—F1A—C17	29.2 (19)	F23A—F21—F21A—C37	−33.1 (13)
F1A—F2—F2A—C17	−23.6 (14)	F23A—F23—F22A—C37	24.6 (9)
C17—F2—F2A—F3	26.8 (11)	C37—F23—F22A—F22	−25.6 (9)
F1A—F2—F2A—F3	3 (2)	F23A—F23—F22A—F22	−1.0 (16)
F3A—F3—F2A—F2	−11 (4)	F21A—F22—F22A—F23	2 (2)
C17—F3—F2A—F2	−34.1 (15)	C37—F22—F22A—F23	29.5 (10)
F3A—F3—F2A—C17	23 (3)	F21A—F22—F22A—C37	−27.6 (16)
F2A—F3—F3A—C17	−18 (2)	F21A—F21—F23A—C37	28.9 (11)
C17—F3—F3A—F1	26.6 (18)	C37—F21—F23A—F23	−19.7 (14)
F2A—F3—F3A—F1	8 (4)	F21A—F21—F23A—F23	9 (2)
F1A—F1—F3A—F3	2 (4)	F22A—F23—F23A—F21	−6 (2)
C17—F1—F3A—F3	−35 (3)	C37—F23—F23A—F21	22.6 (16)
F1A—F1—F3A—C17	37 (2)	F22A—F23—F23A—C37	−28.5 (11)
C11—N2—C1—O1	7.5 (6)	C31—N22—C21—O21	4.1 (6)
C3—N2—C1—O1	−178.0 (3)	C23—N22—C21—O21	176.5 (3)
C11—N2—C1—C7A	−171.5 (3)	C31—N22—C21—C27A	−174.4 (3)
C3—N2—C1—C7A	3.0 (4)	C23—N22—C21—C27A	−2.1 (4)
C1—N2—C3—C3A	13.8 (4)	C21—N22—C23—C23A	17.2 (4)
C11—N2—C3—C3A	−171.4 (3)	C31—N22—C23—C23A	−169.9 (3)
C6—O8—C3A—C3	−167.2 (3)	C26—O28—C23A—C23	−166.9 (3)
C6—O8—C3A—C7A	−54.4 (3)	C26—O28—C23A—C24	60.0 (3)
C6—O8—C3A—C4	58.3 (3)	C26—O28—C23A—C27A	−53.8 (3)
N2—C3—C3A—O8	85.4 (3)	N22—C23—C23A—O28	85.8 (3)
N2—C3—C3A—C7A	−24.7 (3)	N22—C23—C23A—C24	−153.2 (3)
N2—C3—C3A—C4	−152.0 (3)	N22—C23—C23A—C27A	−25.0 (3)
O8—C3A—C4—C5	−32.6 (3)	O28—C23A—C24—C25	−36.2 (3)
C3—C3A—C4—C5	−159.7 (3)	C23—C23A—C24—C25	−161.6 (3)
C7A—C3A—C4—C5	74.4 (3)	C27A—C23A—C24—C25	71.8 (3)
O8—C3A—C4—Br1	85.9 (3)	O28—C23A—C24—Br21	81.9 (3)
C3—C3A—C4—Br1	−41.2 (4)	C23—C23A—C24—Br21	−43.5 (4)
C7A—C3A—C4—Br1	−167.1 (2)	C27A—C23A—C24—Br21	−170.0 (2)
C3A—C4—C5—C6	−3.6 (3)	C23A—C24—C25—C26	0.4 (3)
Br1—C4—C5—C6	−122.6 (2)	Br21—C24—C25—C26	−117.3 (3)
C3A—C4—C5—Br2	−128.3 (2)	C23A—C24—C25—Br22	−123.9 (2)
Br1—C4—C5—Br2	112.7 (2)	Br21—C24—C25—Br22	118.5 (2)
C3A—O8—C6—C7	57.2 (3)	C23A—O28—C26—C27	58.8 (3)
C3A—O8—C6—C5	−59.4 (3)	C23A—O28—C26—C25	−58.6 (3)
C4—C5—C6—O8	38.5 (3)	C24—C25—C26—O28	35.6 (3)
Br2—C5—C6—O8	161.8 (2)	Br22—C25—C26—O28	158.5 (2)
C4—C5—C6—C7	−69.5 (4)	C24—C25—C26—C27	−73.4 (4)
Br2—C5—C6—C7	53.8 (4)	Br22—C25—C26—C27	49.4 (4)
O8—C6—C7—C7A	−36.4 (3)	O28—C26—C27—C27A	−39.5 (3)
C5—C6—C7—C7A	69.2 (3)	C25—C26—C27—C27A	67.9 (4)
O1—C1—C7A—C3A	162.7 (3)	O21—C21—C27A—C23A	167.8 (3)
N2—C1—C7A—C3A	−18.2 (3)	N22—C21—C27A—C23A	−13.6 (4)
O1—C1—C7A—C7	50.5 (5)	O21—C21—C27A—C27	55.6 (5)
N2—C1—C7A—C7	−130.5 (3)	N22—C21—C27A—C27	−125.8 (3)
O8—C3A—C7A—C1	−91.0 (3)	O28—C23A—C27A—C21	−92.9 (3)
C3—C3A—C7A—C1	26.4 (3)	C23—C23A—C27A—C21	23.9 (3)
C4—C3A—C7A—C1	162.1 (3)	C24—C23A—C27A—C21	159.6 (3)
O8—C3A—C7A—C7	31.9 (3)	O28—C23A—C27A—C27	29.7 (3)
C3—C3A—C7A—C7	149.3 (3)	C23—C23A—C27A—C27	146.5 (3)
C4—C3A—C7A—C7	−75.0 (3)	C24—C23A—C27A—C27	−77.8 (3)
C6—C7—C7A—C1	114.7 (3)	C26—C27—C27A—C21	117.8 (3)
C6—C7—C7A—C3A	2.5 (3)	C26—C27—C27A—C23A	5.4 (3)
C1—N2—C11—C12	160.0 (3)	C21—N22—C31—C32	159.3 (3)
C3—N2—C11—C12	−14.1 (5)	C23—N22—C31—C32	−12.5 (5)
C1—N2—C11—C16	−18.8 (5)	C21—N22—C31—C36	−19.9 (5)
C3—N2—C11—C16	167.1 (3)	C23—N22—C31—C36	168.3 (3)
C16—C11—C12—C13	0.2 (6)	C36—C31—C32—C33	0.3 (6)
N2—C11—C12—C13	−178.7 (3)	N22—C31—C32—C33	−178.9 (4)
C11—C12—C13—C14	−0.1 (6)	C31—C32—C33—C34	−0.1 (6)
C12—C13—C14—C15	0.0 (6)	C32—C33—C34—C35	0.4 (6)
C12—C13—C14—C17	−177.8 (4)	C32—C33—C34—C37	−179.8 (4)
C13—C14—C15—C16	0.1 (6)	C33—C34—C35—C36	−0.9 (6)
C17—C14—C15—C16	177.8 (4)	C37—C34—C35—C36	179.3 (4)
C14—C15—C16—C11	0.0 (5)	C34—C35—C36—C31	1.1 (6)
C12—C11—C16—C15	−0.1 (5)	C32—C31—C36—C35	−0.8 (5)
N2—C11—C16—C15	178.7 (3)	N22—C31—C36—C35	178.3 (3)
F3—F3A—C17—F2	48 (3)	F21—F23A—C37—F22	−67.6 (15)
F1—F3A—C17—F2	−101.8 (9)	F23—F23A—C37—F22	94.5 (9)
F3—F3A—C17—F1A	132 (2)	F23—F23A—C37—F21	162.1 (13)
F1—F3A—C17—F1A	−18.0 (9)	F21—F23A—C37—F23	−162.1 (13)
F1—F3A—C17—F3	−150 (2)	F21—F23A—C37—F22A	−142.4 (11)
F3—F3A—C17—F1	150 (2)	F23—F23A—C37—F22A	19.7 (8)
F3—F3A—C17—F2A	20 (2)	F21—F23A—C37—F21A	−31.8 (13)
F1—F3A—C17—F2A	−129.8 (8)	F23—F23A—C37—F21A	130.3 (7)
F3—F3A—C17—C14	−101 (2)	F21—F23A—C37—C34	92.0 (12)
F1—F3A—C17—C14	109.0 (6)	F23—F23A—C37—C34	−105.8 (6)
F2A—F2—C17—F3A	−55.3 (17)	F21A—F22—C37—F23A	62.3 (14)
F1A—F2—C17—F3A	99.9 (10)	F22A—F22—C37—F23A	−95.3 (10)
F2A—F2—C17—F1A	−155.2 (17)	F21A—F22—C37—F21	25.5 (12)
F2A—F2—C17—F3	−34.5 (16)	F22A—F22—C37—F21	−132.0 (8)
F1A—F2—C17—F3	120.7 (9)	F21A—F22—C37—F23	137.3 (11)
F2A—F2—C17—F1	−146.5 (14)	F22A—F22—C37—F23	−20.3 (8)
F1A—F2—C17—F1	8.7 (10)	F21A—F22—C37—F22A	157.6 (13)
F1A—F2—C17—F2A	155.2 (17)	F22A—F22—C37—F21A	−157.6 (13)
F2A—F2—C17—C14	93.5 (15)	F21A—F22—C37—C34	−97.7 (12)
F1A—F2—C17—C14	−111.3 (8)	F22A—F22—C37—C34	104.7 (8)
F1—F1A—C17—F3A	38 (2)	F21A—F21—C37—F23A	−147.4 (13)
F2—F1A—C17—F3A	−125.4 (8)	F23A—F21—C37—F22	130.4 (12)
F1—F1A—C17—F2	163 (2)	F21A—F21—C37—F22	−17.0 (8)
F1—F1A—C17—F3	61 (2)	F23A—F21—C37—F23	17.1 (12)
F2—F1A—C17—F3	−101.5 (8)	F21A—F21—C37—F23	−130.3 (7)
F2—F1A—C17—F1	−163 (2)	F23A—F21—C37—F22A	55.1 (16)
F1—F1A—C17—F2A	149.1 (17)	F21A—F21—C37—F22A	−92.3 (11)
F2—F1A—C17—F2A	−13.9 (9)	F23A—F21—C37—F21A	147.4 (13)
F1—F1A—C17—C14	−87.5 (19)	F23A—F21—C37—C34	−105.0 (12)
F2—F1A—C17—C14	109.5 (6)	F21A—F21—C37—C34	107.6 (7)
F2A—F3—C17—F3A	160 (2)	F22A—F23—C37—F23A	152.5 (11)
F3A—F3—C17—F2	−142 (2)	F22A—F23—C37—F22	27.3 (10)
F2A—F3—C17—F2	18.5 (9)	F23A—F23—C37—F22	−125.3 (8)
F3A—F3—C17—F1A	−58 (2)	F22A—F23—C37—F21	141.3 (10)
F2A—F3—C17—F1A	101.8 (9)	F23A—F23—C37—F21	−11.3 (8)
F3A—F3—C17—F1	−31 (2)	F23A—F23—C37—F22A	−152.5 (11)
F2A—F3—C17—F1	129.1 (8)	F22A—F23—C37—F21A	63.3 (13)
F3A—F3—C17—F2A	−160 (2)	F23A—F23—C37—F21A	−89.2 (12)
F3A—F3—C17—C14	91 (2)	F22A—F23—C37—C34	−97.9 (10)
F2A—F3—C17—C14	−109.0 (7)	F23A—F23—C37—C34	109.6 (7)
F1A—F1—C17—F3A	−144 (2)	F23—F22A—C37—F23A	−26.7 (11)
F1A—F1—C17—F2	−17 (2)	F22—F22A—C37—F23A	124.9 (8)
F3A—F1—C17—F2	127.0 (8)	F23—F22A—C37—F22	−151.6 (10)
F3A—F1—C17—F1A	144 (2)	F23—F22A—C37—F21	−58.8 (13)
F1A—F1—C17—F3	−130.2 (19)	F22—F22A—C37—F21	92.8 (11)
F3A—F1—C17—F3	13.7 (10)	F22—F22A—C37—F23	151.6 (10)
F1A—F1—C17—F2A	−40 (2)	F23—F22A—C37—F21A	−136.9 (9)
F3A—F1—C17—F2A	103.5 (10)	F22—F22A—C37—F21A	14.6 (9)
F1A—F1—C17—C14	105.3 (18)	F23—F22A—C37—C34	101.2 (9)
F3A—F1—C17—C14	−110.7 (7)	F22—F22A—C37—C34	−107.2 (6)
F2—F2A—C17—F3A	137.6 (14)	F22—F21A—C37—F23A	−134.3 (11)
F3—F2A—C17—F3A	−9.1 (11)	F21—F21A—C37—F23A	20.0 (8)
F3—F2A—C17—F2	−146.7 (15)	F21—F21A—C37—F22	154.3 (12)
F2—F2A—C17—F1A	24.9 (17)	F22—F21A—C37—F21	−154.3 (12)
F3—F2A—C17—F1A	−121.8 (9)	F22—F21A—C37—F23	−61.4 (14)
F2—F2A—C17—F3	146.7 (15)	F21—F21A—C37—F23	92.9 (10)
F2—F2A—C17—F1	44.6 (17)	F22—F21A—C37—F22A	−21.3 (13)
F3—F2A—C17—F1	−102.1 (9)	F21—F21A—C37—F22A	133.0 (8)
F2—F2A—C17—C14	−100.8 (14)	F22—F21A—C37—C34	99.9 (10)
F3—F2A—C17—C14	112.4 (7)	F21—F21A—C37—C34	−105.8 (6)
C15—C14—C17—F3A	35.6 (11)	C35—C34—C37—F23A	83.5 (11)
C13—C14—C17—F3A	−146.7 (10)	C33—C34—C37—F23A	−96.2 (11)
C15—C14—C17—F2	−118.1 (9)	C35—C34—C37—F22	−113.7 (8)
C13—C14—C17—F2	59.6 (10)	C33—C34—C37—F22	66.5 (9)
C15—C14—C17—F1A	157.9 (13)	C35—C34—C37—F21	124.9 (8)
C13—C14—C17—F1A	−24.4 (14)	C33—C34—C37—F21	−54.9 (9)
C15—C14—C17—F3	6.2 (11)	C35—C34—C37—F23	7.9 (8)
C13—C14—C17—F3	−176.1 (10)	C33—C34—C37—F23	−171.9 (7)
C15—C14—C17—F1	125.6 (8)	C35—C34—C37—F22A	−40.1 (13)
C13—C14—C17—F1	−56.8 (9)	C33—C34—C37—F22A	140.1 (12)
C15—C14—C17—F2A	−81.9 (13)	C35—C34—C37—F21A	−157.2 (11)
C13—C14—C17—F2A	95.8 (13)	C33—C34—C37—F21A	23.0 (12)

### Hydrogen-bond geometry (Å, º)

*Cg*5 and *Cg*10 are the centroids of the C11–C16 and C31–C36 rings, respectively.

**Table:**

*D*—H···*A*	*D*—H	H···*A*	*D*···*A*	*D*—H···*A*
C5—H5*A*···O8^i^	0.98	2.57	3.342 (4)	135
C7—H7*A*···Br2	0.97	2.82	3.300 (4)	112
C16—H16*A*···O1	0.93	2.26	2.853 (4)	121
C27—H27*A*···Br22	0.97	2.78	3.259 (4)	111
C36—H36*A*···O21	0.93	2.28	2.856 (5)	120
C7*A*—H7*AA*···*Cg*10^i^	0.98	2.94	3.741 (4)	139
C27*A*—H27*C*···*Cg*5^i^	0.98	2.97	3.924 (4)	166

Symmetry code: (i) −*x*+1, −*y*+1, −*z*+1.
